# Addition of carboplatin-gemcitabine as second-line neoadjuvant chemotherapy in non-responsive locally advanced breast cancer patients to standard neoadjuvant chemotherapy and evaluation of factors affecting response: a randomized controlled trial

**DOI:** 10.1186/s12885-020-07652-0

**Published:** 2021-01-11

**Authors:** Dena Firouzabadi, Amirreza Dehghanian, Alireza Rezvani, Laleh Mahmoudi, Abdolrasoul Talei

**Affiliations:** 1 Department of Clinical Pharmacy, School of Pharmacy, Shiraz University of Medical Sciences, Shiraz, Iran; 2 Trauma Research Center, Shiraz University of Medical Sciences, Shiraz, Iran; 3 Molecular Pathology and Cytogenetics Section, Department of Pathology, School of Medicine, Shiraz University of Medical Sciences, Shiraz, Iran; 4 Hematology Research Center, Shiraz University of Medical Sciences, Shiraz, Iran; 5 Hematology and Medical Oncology Department, School of Medicine, Shiraz University of Medical Sciences, Shiraz, Iran; 6 Breast Diseases Research Center, Shiraz University of Medical Sciences, Shiraz, Iran

**Keywords:** Locally advanced breast cancer, Pathologic complete response, Neoadjuvant chemotherapy, ki67, Lymph node, Tumor-infiltrating lymphocytes, Carboplatin, Gemcitabine

## Abstract

**Background:**

Neoadjuvant chemotherapy (NACT) is the prime approach to the management of locally advanced breast cancer (LABC). Influenced by different factors such as pathologic tumor characteristics, hormone receptor status, HER2 and proliferation marker expressions, response to therapy cannot be easily predicted. Pathologic complete response (pCR) has been considered as an endpoint to NACT; however, pCR rates have been unsatisfactory in such patients. In this randomized trial, we studied the efficacy of carboplatin/gemcitabine as second-line NACT while evaluating the impact of different factors affecting response.

**Methods:**

In this randomized controlled trial, 52 clinically non-responsive (confirmed by palpation and/or ultrasonography) LABC patients to 4 cycles of doxorubicin/cyclophosphamide followed by 4 cycles of paclitaxel ± trastuzumab were randomly allocated to two groups. “Control” group underwent breast surgery and were further evaluated for pCR (ypT0/is ypN0). “Intervention” group received 2 cycles of carboplatin/gemcitabine and patients were further evaluated for pCR following surgery.

**Results:**

In a total of 52 patients, pCR rate was 30.7%. pCR and response rate in lymph nodes were higher in carboplatin/gemcitabine recipients (32% vs 29.7 and 44% vs 40.7% respectively), however differences were insignificant. In both the “intervention” group and total study population, most pCR cases were of the hormone receptor (HR)+/HER2+ subtype (87.5% and 75% respectively). HER2 positivity, ki67 expression, lower extent of ER positivity, higher tumor grade and tumor-infiltrating lymphocyte (TIL) lead to higher pCR rates. Adverse events following addition of carboplatin/gemcitabine were mostly hematologic and none required hospitalization. Anemia was the most common grade 3 adverse event observed. No grade 4 toxicity was evident.

**Conclusion:**

Although the proposed carboplatin/gemcitabine combination could not improve pCR rates as expected, probability of immune activation following use of carboplatin in achieving response to NACT may be considered. Accounting for the highest number of pCR cases in the “intervention” group, the HR+/HER2+ subtype with high TILs may be considered as most responsive to the proposed regimen in this study. It is noteworthy that the proposed combination imposed minimal toxicity.

**Trial registration:**

This trial was prospectively registered in IRCT.ir (IRCT2017100136491N1). Date of registration: 19 November 2017.

## Background

Locally advanced breast cancer (LABC) is a subset of breast cancer defined as tumors larger than 5 cm with regional lymphadenopathy, or tumors with direct extension to the chest wall and/or skin or tumors with regional lymphadenopathy regardless of the tumor stage [1]. This type of breast cancer is not scarce constituting around 50% of breast cancer patients in developing countries and 20% in developed ones [2]. Its advanced stage at diagnosis with high probability of relapse and eventual death brings about the importance of approach to LABCs [3]. Neoadjuvant chemotherapy (NACT) is the most common modality of treatment used for this type of breast cancer [4]. NACT can have several benefits including reduction in tumor size and need for nodal operation, omission of micro metastasis and increase in breast conservation rates and also possibility of resection of inoperable mass. Also it can provide an early estimation of a patient’s response to treatment and overall outcome [5, 6]. The endpoint to achieving response to therapy is pathologic complete response (pCR) considered as a substantial element predicting long term outcome in breast cancer patients [7]. pCR has been diversely defined as the absence of residual tumor in solely the breast or both the breast and axillary nodes. Also the presence or absence of ductal carcinoma in situ (DCIS) has been another point of diversity in its definition [8]. However the US Food and Drug Administration (FDA) reported that no residual tumor in both breast and axillary nodes (ypT0/is ypN0) is associated with better outcome regardless of presence or absence of DCIS [9]. Residual cancer burden (RCB) has also been introduced as a new approach to evaluating degree of pathologic response to NACT [10]. Despite an overall poor prognosis, LABCs are chemo-sensitive. Different chemotherapy regimens have been used to date in order to achieve better response rates. Anthracycline-based regimens have been stated as being superior to other regimens and have been selected as regimens of choice in combination with cyclophosphamide and addition of a taxane agent thereafter [11–14]. Taxane derivatives have been found effective in breast cancers with lymph node involvement [15]. The benefit of either sequential or in-combination administration of the taxane agent is yet controversial however both methods have shown equal survival rates [3]. NACT with the intention of achieving pCR has been associated with poor response rates in LABC patients ranging from 3 to 30% [3, 16–18].

Hormone receptor status (estrogen and progesterone), human epidermal growth factor receptor 2 (HER2) and nuclear protein ki67 known as a proliferation marker, have been associated with breast cancer prognosis and presented as predictive factors in response to NACT in LABC [16, 19].

Tumor-infiltrating lymphocytes (TILs) have been proposed as an immune mechanism in defeating cancer progression. The amount of lymphocytes gathered in tumoral/stromal cells has been suggested to be predictive of response to NACT [20].

Considering the low pCR rates in LABC following NACT and the positive prognostic impact of pCR on such patients’ outcome, in this randomized clinical trial we decided to evaluate the efficacy of a second line NACT in clinically non-responsive patients to a first line anthracycline-taxane based NACT and study the effect of probable factors affecting response. Carboplatin has been effective in increasing pCR rates in some subtypes of breast cancer [21, 22]. Gemcitabine has also shown promising results in approach to LABC with minimal toxicity [23, 24]. Therefore in this study the combination of the two mentioned chemotherapy agents was used as second line NACT with the intension of improving pCR rates.

## Methods

This experimental, prospective, parallel randomized controlled trial (RCT) took place to evaluate the efficacy of addition of carboplatin/gemcitabine combination as second-line NACT in LABC patients.

### Patient selection

Study population was gathered from 2 major referral teaching clinics. All female patients 18 years or older, newly diagnosed with LABC according to NCCN guidelines [25] and histologic confirmation with no prior treatment history were eligible for taking part in this study.

Patients with confirmed LABC diagnosis, having sufficient hematological, kidney, hepatic and cardiac function (ANC ≥ 1.5 × 10^9^/l; platelets ≥100 × 10^9^/l; hemoglobin ≥8 g/dl; serum bilirubin < 1.5 upper limit of normal; serum transaminase < 2.5 ULN; serum creatinine within normal range; left ventricular ejection fraction (LVEF) > 50%) were included in the study. Women older than 60 and younger than 18 years of age, patients with presence of metastatic or inflammatory breast cancer, history of previous chemotherapy (other than first line NACT given by our team), radiotherapy or surgery for the treatment of breast cancer, presence of positive pregnancy tests, active infection, HBV, HCV and HIV infection were excluded from the study.

### Treatment plan

LABC patients clinically non-responsive evaluated by palpation and/or ultrasound sonography (US) 2–3 weeks following completion of first line NACT including combination of doxorubicin 60 mg/m^2^ and cyclophosphamide 600 mg/m^2^ for 4 cycles every 2 weeks followed by paclitaxel 175 mg/m^2^ ± trastuzumab (in HER2 positive patients) for 4 cycles every 2 weeks, were randomly allocated to two “control” and “intervention” groups. The “control” group patients were assigned to surgical removal of the mass after termination of first line NACT, and the “intervention” group patients were allocated to receiving two extra cycles of NACT as second line NACT consisting of carboplatin target AUC 5 and gemcitabine 1 g/m^2^ days 1,8 (2 cycles every 3 weeks). The “intervention” group patients were thereafter scheduled for breast surgery.

### Outcome

The primary objective of this randomized controlled study was to evaluate pCR rate for response to NACT in both study groups. pCR rates were assessed by an expert pathologist after breast surgery was performed and post-surgical biopsy specimens were ready for evaluation. pCR was defined as no residual tumor in both breast and axillary nodes (ypT0/is ypN0). RCB was also calculated for each post-surgical specimen according to primary tumor bed, remaining tumor cellularity, presence of DCIS, number of involved lymph nodes and their largest dimension. RCB 0 indicates no residual tumor (pCR), RCB I indicates near to complete response, RCB II shows partial response and RCB III represents the tumor as chemoresistant [10].

### Randomization

Permutated block randomization was performed in order to distribute patients into the two assigned groups. A block size of 4 was selected. The number of blocks used for assessment of randomization was 15, considering probable patient losses during the study. For a block size of 4, 6 permutations can be considered for two study groups (A and B). For distribution of the mentioned allocations to each block, a random number table was used. Allocations sequence was implemented by the clinical pharmacist, patients were enrolled by the hematologist-oncologist and participants were assigned to each study group by the clinical pharmacist following the sequence acquired using the random number table (BAAB BAAB AABB AABB BBAA BAAB ABBA AABB AABB ABAB ABAB BBAA BBAA ABBA BBAA).

### Blinding

In this study, patients, the pathologist evaluating response to NACT and the statistical analyst were blind to allocation of subjects to either groups of treatment.

### Ethics committee approval

This study was conducted following the declaration of Helsinki regarding ethical principles for medical research and approved by Shiraz University of Medical Sciences ethics committee (approval code: IR.SUMS.REC.1396.S372). The project was also registered in the Iranian registry of clinical trials (IRCT) and the unique allocated code for this research project was obtained (IRCT2017100136491N1).

Written informed consent was obtained from all patients taking part in this study and patients were all made aware that they could leave the study process at any time if desired.

### Statistical analysis

After gathering all related data, SPSS v25 software was used to analyze the relevant data. Independent sample’s t-test was used for comparing quantitative variables between the groups. Mann-Whitney test was used for evaluation of non-normal parameters. Pearson chi square and Fisher exact tests were used to compare the qualitative variables between the groups. Logistic regression was performed to analyze the impact of different tumor and patient characteristics in achieving pCR. *P* values ≤0.05 were considered statistically significant.

### LABC diagnosis and baseline evaluation of patients

Diagnostic mammography and ultra-sonography were performed for breast cancer suspicious patients. Core needle biopsy was obtained for confirmation of diagnosis. Bone scan, computed topography (CT) and in some cases magnetic resonance imaging (MRI) were performed to rule out probability of metastasis.

### Immunohistochemical analysis

Hormone receptors (HR), estrogen and progesterone (ER, PR respectively), HER2 and ki67 expressions were evaluated using Immunohistochemical (IHC) assay on core needle biopsy samples of the breast mass. Specific monoclonal antibodies, 1D5 (ER), SP2 (PR), CB11 (HER2) and SP6 (ki67) were obtained from Biocare Medical, Ca, USA and used for the IHC assays. Paraffin embedded blocks of breast tissue were used for the preparation of slides. Further deparaffinization, antigen retrieval, antigen-antibody reaction, secondary antibody reaction with horseradish protein (HRP) and at last immunostaining were performed. Interpretation of ER, PR, HER2 and ki67 expression was performed by an expert pathologist using the introduced cut points in literature. Cutoff points of 1, 10 and 20% were respectively used for ER/PR, HER2 and ki67 positivity [26–28]. In cases of equivocal HER2 condition, fluorescence in situ hybridization (FISH) test was performed.

Molecular subtyping of breast tumors was performed based on hormonal status, HER2 and ki67 expressions [29]. Accordingly patients were categorized to 4 different subtypes: HR+/HER2-, HR+/HER2+, HR−/HER2+ and HR−/HER2- known as triple negative breast cancer (TNBC).

### TIL evaluation

TIL was estimated using slides prepared from core needle biopsy specimens of breast tissue. Calculation was based on percentage of tumoral cell lymphocyte infiltration. Cut point used for marking a specimen as high infiltration was selected 10% according to previously published data [20].

### Pathology image acquisition

Pathology images were acquired using an Olympus BX51 light microscope (Tokyo, Japan) fitted with a 5-M pixel high resolution color camera (Olympus, DP25). DP2-BSW software was used for the extraction of images. Images were obtained with a 2560 × 1920 pixel resolution and no downstream processing or enhancement in the resolution was done.

### TNM staging

Residual tumor was staged both anatomically and prognostically according to the American Joint Committee on Cancer (AJCC 8th edition) [30] classification after receiving NACT. Anatomical staging is based on involvement of tissue (T), node (N) and presence of metastasis (M), however prognostic classification incorporates, tumor grade, hormonal and HER2 status along with the mentioned T, N and M. It must be explained that non-pCR cases with residual tumoral tissue/lymph node were further staged and stage 0 stated, accounts for cases with residual Tis (insitu) with no residual lymph node (N0).

### Evaluation of NACT related adverse events

Evaluation of adverse events following chemotherapy was performed based on the Common Terminology Criteria for Adverse Events (CTCAE) following completion of carboplatin/gemcitabine cycles.

## Results

**Fig. 1:**
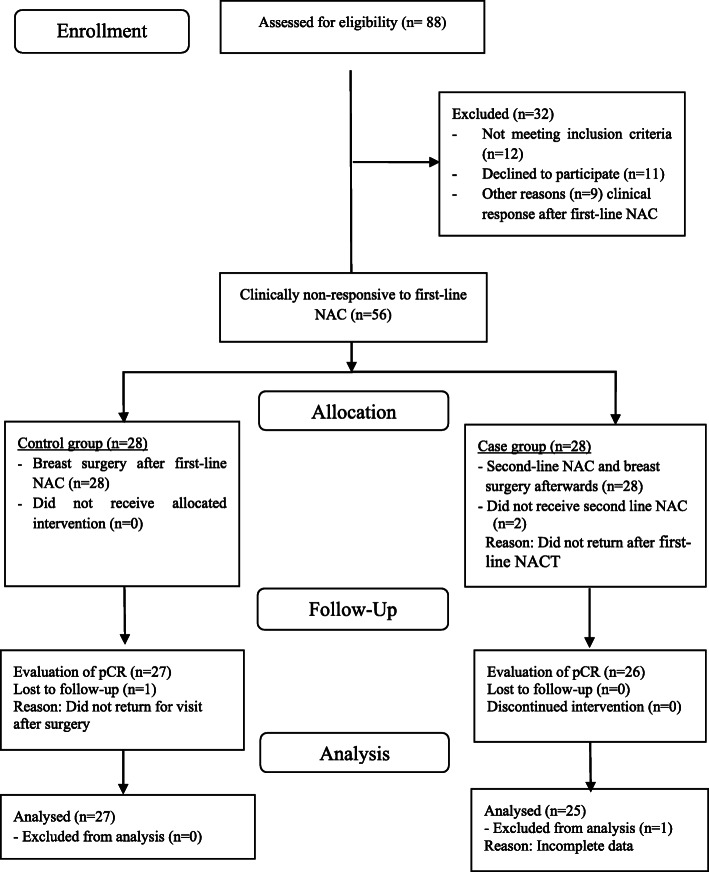
Consort flow diagram for patient allocation to each group

From October 2017 to September 2019, 137 breast cancer patients were evaluated for eligibility. Eighty-eight patients were suspicious for LABC diagnosis. As shown in flow diagram of this study process (Fig. 1), at last 56 LABC patients clinically non-responsive to first-line anthracycline- and taxane-based NACT, were randomized to either control or intervention groups. Detailed flow diagram of patient allocation is elaborated in Fig. 1.

Patients’ baseline demographic characteristics, clinical and pathological features of the tumor are elaborated in Table 1.

**Table 1:** Demographic, clinical and pathological features of study population

Demographic data	Group		*P value*
	Control *(n = 27)*	Intervention *(n = 25)*	
**Age** (mean ± SD)	45.0 ± 8.36	41.0 ± 8.61	0.099
**Weight** (mean ± SD)	68.0 ± 9.52	70.0 ± 9.71	0.45
**BMI** (mean ± SD)	27.4 ± 4.37	27.7 ± 4.03	0.78
**BSA** (mean ± SD)	1.7 ± 0.1	1.7 ± 0.13	0.35
**Positive family history** n (%)	8 (29.6)	6 (24.0)	0.65
**Regular Menstruation** n (%)	20 (74.1)	21 (84.0)	0.38
**Married** n (%)	26 (96.0)	22 (88.0)	0.34
**Parity** (mean±SD)	2.8 ± 1.96	1.88 ± 1.36	0.069
**Primary tumor size mm** (mean±SD)	31.3 ± 19.54	41.2 ± 32.78	0.364
**Initial T-stage** n (%)			
TI	7 (25.9)	4 (16.0)	0.611
T2	6 (22.2)	9 (36.0)	
T3	4 (14.8)	5 (20.0)	
T4	10 (37.0)	7 (28.0)	
**Tumor grade** n (%)			
I	6 (22.2)	3 (12.0)	0.657
II	13 (48.1)	13 (52.0)	
III	8 (29.6)	9 (36.0)	
**Node involvement** n (%)	21 (77.7)	22 (88.0)	0.469
**Hormonal status**			
ER+ n (%)	21 (85.0)	23 (84.0)	1.00
Extent of positivity (mean±SD)	74.3 ± 25.1	59.9 ± 33.9	0.104
PR+ n (%)	19 (70.0)	14 (56.0)	0.28
Extent of positivity (mean±SD)	48.7 ± 32.7	46.78 ± 32.7	0.869
**HER2+** n (%)	10 (37.0)	8 (32.0)	0.43
**ki67+**^a^ n (%)	15 (55.5)	19 (76.0)	0.122
**ki67** (mean±SD)	34.8 ± 27.8	37.2 ± 23.7	0.742
**Molecular Subtype**			
HR+/HER2-	12 (44.4%)	9 (36%)	0.98
HR+/HER2+	11 (40.1%)	12 (48%)	
HR-/HER2+	2 (7.4%)	2 (8%)	
TNBC	2 (7.4%)	2 (8%)	
**TIL**^b^ n (%)			
+	13 (48.1)	10 (40.0)	0.554
-	14 (51.9)	15 (60.0)	

*BMI* Body mass index, *BSA* Body surface area, *ER* Estrogen receptor, *PR* Progesterone receptor, *NACT* neoadjuvant chemotherapy, *TNBC* Triple negative breast cancer, *TIL* Tumor-infiltrating lymphocytes

^a^Cut-off for ki67 positivity was 20%

^b^Cut-off for TIL was 10%

As shown in Table 1, no difference with regard to demographic data and pathologic tumor characteristics was observed in between the two groups after randomization. ER, PR, HER2 and ki67 status of the study population in both study groups are shown.

In Table 1, distribution of the study population in four different breast cancer subtypes is observed. HR+ tumors accounted for the majority of tumors in our study population.

### Efficacy

The endpoint to NACT in our study was introduced as pCR. pCR rates were 29.6 and 32% respectively for “control” and “intervention” groups; however, no significant difference was observed between the two arms with regard to pCR (*P* value 0.853). DCIS was present in 18.5 and 28% of “control” and “intervention” groups respectively showing no statistically significant difference between the groups (*P* value 0.42). Response rates in the tissue only and lymph nodes only have also been elaborated in both groups in Table 2. Degree of pathologic response to NACT based on RCB scoring is elaborated in Table 3. As shown, 59.6% of the overall study population proved to respond to therapy (RCB 0, I and II) although 40.4% were mainly chemoresistant regardless of the NACT regimen used.

**Table 2:** pCR and response rates in tissue or only lymph nodes in “control” and “intervention” groups

Response	group		*P* value
	control *n = 27* n (%)	intervention *n = 25* n (%)	
pCR	8 (29.6%)	8 (32%)	0.853
Breast tissue	14 (51.9%)	12 (48%)	0.781
Lymph nodes	11 (40.7%)	11 (44%)	0.812

*pCR* pathologic complete response

**Table 3:** Pathologic response to NACT according to Residual Cancer Burden (RCB) scoring [10].

Group	RCB			
	0 n (%)	I n (%)	II n (%)	III n (%)
Control *n = 27*	8 (29.6)	2 (7.4)	6 (22.2)	11 (40.7)
Intervention *n = 25*	8 (32.0)	2 (8.0)	5 (20.0)	10 (40.0)
Total *n = 52*	16 (30.8)	4 (7.7)	11 (21.2)	21 (40.4)

Generally different factors have long been studied in affecting pCR rates. The probable association of age, BMI and primary tumor size with pCR rates in both arms is demonstrated in Table 4. As shown, older age was associated with achieving pCR in the “intervention” group. Smaller primary tumor size was also more prone to achieving response in the total study population; however, difference was close to being significant (*P* value 0.065).

**Table 4:** Association of age, BMI and primary tumor size with achieving pCR

Factor	group	response to NAC		*P* value
		pCR	non-pCR	
Age (mean ± SD)	Control *n* = 27	41.37 ± 5.47	46.52 ± 9.01	0.147
	Intervention *n* = 25	46.12 ± 5.56	38.65 ± 8.86	*0.04*
	Total *n* = 52	43.75 ± 5.87	42.8 ± 9.67	0.655
BMI (mean ± SD)	Control *n* = 27	28.05 ± 3.73	27.1 ± 4.69	0.618
	Intervention *n* = 25	25.85 ± 3.09	27.17 ± 4.39	0.344
	Total *n* = 52	28.45 ± 3.34	27.14 ± 4.49	0.301
Primary tumor size (mean ± SD)	Control *n* = 27	23.8 ± 12.65	34.45 ± 21.31	0.205
	Intervention *n* = 25	28.12 ± 19.15	47.29 ± 36.42	0.178
	Total *n* = 52	26 ± 15.83	40.5 ± 29.7	0.065

*BMI* body mass index, *pCR* pathologic complete response

Association of different molecular subtypes of breast cancer, ER, PR, HER2 and ki67 expression with achieving pCR was evaluated and demonstrated in Table 5. As shown, HR+/HER2+ subtype was associated with better pCR rates in the intervention group and total study population (*P* value 0.014 and 0.002 respectively) attributing highest number of pCR cases to this molecular subtype in both mentioned groups (87.5 and 75% respectively). HER2 negativity was also associated with not achieving pCR in the “control” group and total study population (*P* value 0.027 and 0.005 respectively). Proliferation marker ki67 was also associated with better response rates in the overall study population (*P* value 0.025). Data regarding logistic regression analysis of different variables affecting pCR is brought in Table 6. As shown high tumor grade (III), HER2 and ki67 expression, TILs and HR+/HER2+ subtype were variables relating to achieving pCR in this study population.

**Table 5:** Univariate analysis of pCR according to breast cancer molecular expressions and TILs

Group	pCR	Covariate				*P* value
		***Molecular subtypes***				
		***HR+/HER2-***	***HR+/HER2+***	***HR-/HER2+***	***TNBC***	
Intervention		*n = 9*	*n = 12*	*n = 2*	*n = 2*	
	**+**	0	7 (58.3)	0	1 (50.0)	*0.014*
	**-**	9 (100.0)	5 (41.7)	2 (100.0)	1 (50.0)	
Control		*n = 12*	*n = 11*	*n = 2*	*n = 2*	
	**+**	1 (8.3)	5 (45.5)	1 (50.0)	1 (50.0)	0.125
	**-**	11 (91.7)	6 (54.5)	1 (50.0)	1 (50.0)	
Total		*n = 21*	*n = 23*	*n = 4*	*n = 4*	
	**+**	1 (4.8)	12 (52.2)	1 (25.0)	2 (50.0)	*0.002*
	**-**	20 (95.2)	11 (47.8)	3 (75.0)	2 (50.0)	
		***ER Status***				
		***positive***		***negative***		
Intervention		*n = 21*		*n = 4*		
	**+**	7 (33.3)		1 (25.0)		1.00
	**-**	14 (66.7)		3 (75.0)		
Control		*n = 23*		*n = 4*		
	**+**	6 (26.1)		2 (50.0)		0.558
	**-**	17 (73.9)		2 (50.0)		
Total		*n = 44*		*n = 8*		
	**+**	13 (29.5)		3 (37.5)		0.689
	**-**	31 (70.5)		5 (62.5)		
		***PR Status***				
		***positive***		***negative***		
Intervention		*n = 14*		*n = 11*		
	+	4 (28.6)		4 (36.3)		1.00
	-	10 (71.4)		7 (63.6)		
Control		*n = 19*		*n = 8*		
	+	5 (26.3)		3 (37.5)		0.658
	-	14 (73.7)		5 (62.5)		
Total		*n = 33*		*n = 19*		
	+	9 (27.3)		7 (36.8)		0.472
	-	24 (72.7)		12 (63.1)		
		***HER2 Status***				
		***positive***		***negative***		
Intervention		*n = 10*		*n = 15*		
	**+**	5 (50.0)		3 (20.0)		0.194
	**-**	5 (50.0)		12 (80.0)		
Control		*n = 8*		*n = 19*		
	**+**	5 (62.5)		3 (15.8)		*0.027*
	**-**	3 (37.5)		16 (84.2)		
Total		*n = 18*		*n = 34*		
	**+**	10 (55.5)		6 (17.6)		*0.005*
	**-**	8 (44.4)		28 (82.3)		
		***ki67 status***				
		***positive***		***negative***		
Intervention		*n = 19*		*n = 6*		
	**+**	8 (42.1)		0 (0)		0.129
	**-**	11 (57.9)		6 (100.0)		
Control		*n = 15*		*n = 12*		
	**+**	6 (40.0)		2 (16.7)		0.236
	**-**	9 (60.0)		10 (83.3)		
Total		*n = 34*		*n = 18*		
	**+**	14 (41.2)		2 (11.1)		*0.025*
	**-**	20 (58.8)		16 (88.9)		
		***TILS***				
		***positive***		***negative***		
Intervention		*n = 10*		*n = 15*		
	**+**	6 (60.0)		2 (13.3)		*0.028*
	**-**	4 (40.0)		13 (86.7)		
Control		*n = 13*		*n = 14*		
	**+**	5 (38.5)		3 (21.4)		0.420
	**-**	8 (61.5)		11 (78.6)		
Total		*n = 23*		*n = 29*		
	**+**	11 (47.8)		5 (17.2)		*0.018*
	**-**	12 (52.2)		24 (82.7)		

*TNBC* triple negative breast cancer, *pCR* pathologic complete response, *ER* estrogen receptor, *PR* progesterone receptor, *TILs* Tumor-infiltrating lymphocytes

Note: **+** stands for pCR, - stands for non-pCR

**Table 6:** Impact of various independent variables on achieving pCR

Variable	*Univariate analysis*		*Multivariate analysis*	
	*P* value	OR (95% CI)	*P* value	OR (95% CI)
Primary tumor size	0.083	1.032 (0.996–1.07)	0.241	01.031 (0.98–1.085)
High tumor grade (III)	*0.049*	3.75 (1.003–14.02)	*0.025*	0.015 (0.0–0.585)
HER2 positivity	*0.007*	0.171 (0.48–0.617)	*0.02*	0.055 (0.005–0.636)
Ki67 positivity	*0.037*	0.179 (0.035–0.903)	*0.018*	0.028 (0.001–0.548)
TILs	*0.022*	0.227 (0.064–0.80)	*0.015*	0.058 (0.006–0.58)
NACT (Carboplatin+Gemcitabine)	0.853	0.895 (0.275–2.91)	0.518	1.94 (0.26–14.46)
Molecular subtype				
HR+/HER2+	*0.005*	0.045 (0.005–0.399)	*0.026*	0.048 (0.003–0.69)
HR−/HER2+	0.218	0.149 (0.007–3.078)	0.368	0.158 (0.003–8.831)
HR−/HER2-	*0.036*	0.050 (0.003–0.824)	0.452	0.244 (0.006–9.628)

*NACT* Neoadjuvant chemotherapy, *TIL* Tumor-infiltrating lymphocytes, *HR* Hormone receptor, *OR* Odd Ratio, *CI* Confidence Interval

Aside from significant associations demonstrated in Table 5, higher extents of ER expression showed significant association with not achieving pCR in the overall study population regardless of the NACT regimen used (*P* value 0.005). High tumor grade (III) was also associated with better pCR rates in our study population (*P* value 0.043). Majority of pCR cases in both “intervention” and “control” groups had been marked with high tumor grades (50 and 63.5% respectively). TILs was also evaluated as a probable immune response in cancerous cells and significantly improved pCR rates in the “intervention” group and total study population (*P* value 0.028 and 0.018 respectively) (Table 5).

Residual tumor TNM staging is elaborated in Table 7.

**Table 7:** Residual tumor TNM staging according to AJCC classification

Group	TNM Definition	Stage							
		0	IA	IB	IIA	IIB	IIIA	IIIB	IIIC
Control n (%)	*Anatomical*	0	2 (7.4)	3 (11.0)	8 (29.6)	2 (7.4)	0	2	2 (7.4)
	*Prognostic*	0	2 (7.4)	0	7 (25.9)	2 (7.4)	5 (18.5)	1 (3.7)	2 (7.4)
Intervention n (%)	*Anatomical*	2 (8.0)	1 (4.0)	2 (8.0)	6 (24.0)	0	3 (12.0)	5 (20.0)	0
	*Prognostic*	2 (8.0)	1 (4.0)	0	4 (16.0)	1 (4.0)	8 (32.0)	0	3 (12.0)

*AJCC* American joint committee on cancer

**Fig. 2:**
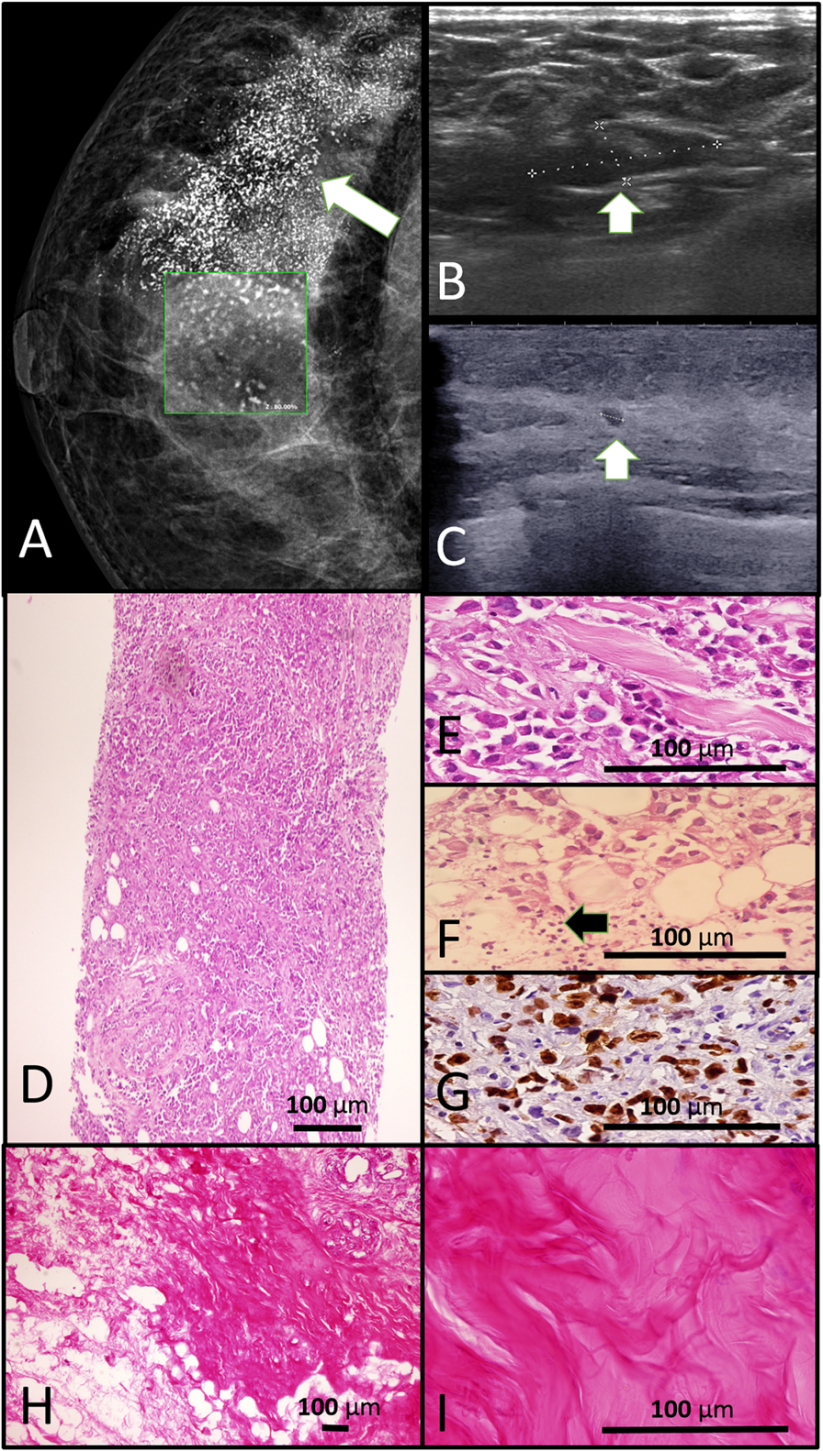
**a** Mammography of the left breast of a patient in the case group in standard craniocaudal (CC) view shows large irregular mass (arrow) with multiple micro calcifications (inlet: X80). **b**, **c** In the same patient, sonography evaluation of the mass before (**b**) and after (**c**) neoadjuvant chemotherapy regimen shows significant decrease in the size of the mass (arrows) without complete clinical response. **d**, **e** Histopathological evaluation of the tru-cut biopsy of the same patient shows high cellular tumor with Nottingham grade III/III (H & E staining X100, X400 respectively). **f** Tumor cells with intratumoral infiltrating lymphocytes (TILs) as are shown by arrow (H & E staining. X400). **g** Immunohistochemical staining of the pre-treatment biopsy for ki-67 antibody shows high proliferative index (X400). **h**, **i** Histopathological evaluation of the surgical specimen of the same patient shows fibrotic tumor bed with no residual tumor following second-line neoadjuvant chemotherapy (Pathologic complete response, Residual cancer burden:0) (H & E staining. X40, X400 respectively)

Baseline diagnostic mammography, ultrasonography, H&E stained tru-cut breast biopsy, IHC stained ki67 expression in tumoral cells, tumoral infiltrating lymphocytes (TILs), post NACT ultrasonography and pCR in breast tissue following second line NACT have been demonstrated in Fig. 2.

### Safety

Adverse events following the addition of carboplatin/gemcitabine as the new proposed second-line NACT were investigated according to CTCAE. Adverse events were mainly hematologic. Anemia, thrombocytopenia, leukopenia and rise in ALT were higher in the “intervention” group. However, all adverse events were of low grade and there was no need for hospital admission and in-patient management. A detailed grading of the adverse events in the intervention group is demonstrated in Table 8.

**Table 8:** Adverse events following addition of carboplatin/gemcitabine combination as second-line NACT in the “intervention” group

Adverse event	Grade 1	Grade 2	Grade 3	Grade 4
Anemia	10 (40)	–	10 (40)	–
ALT rise	10 (40)	7 (28)	1 (4)	–
AST rise	8 (32)	2 (8)	–	–
ALP rise	5 (20)	–	–	–
Neutropenia (↓ANC)	6 (24)	7 (28)	3 (12)	–
Leukopenia	13 (52)	4 (16)	2 (8)	–
Thrombocytopenia	10 (40)	1 (4)	1 (4)	–
Oral mucositis	1 (4)	–	–	–

*ALT* alanine amino transferase, *AST* aspartate amino transferase, *ALP* alkaline phosphatase, *ANC* absolute neutrophil count

## Discussion

In this study we decided to add the combination of carboplatin and gemcitabine as second-line NACT to a first-line anthracycline based NACT followed by paclitaxel in order to improve pathologic response rates. Incomplete response and residual invasive tumor following NACT in LABC patients has proven to worsen overall outcome and survival. And on the contrary, pCR has been introduced to have prognostic value in the patients’ overall outcome [7]. Although promising approaches to adjuvant treatment of non-responsive LABC to NACT has been proposed and has proven to be successful, the value of achieving pCR through pre-surgical chemotherapy (NACT) without doubt has not been underestimated. Anthracycline based regimens have been established as regimens of choice in patients with no serious comorbid cardiac disease and have proven to have overall benefit in such patients compared to more traditional regimens. Addition of a taxane derivative to the mentioned regimens either concomitantly or sequentially has been also found beneficial [12, 14]. As it has been proposed in very recent guidelines to continue additional systemic therapy in non-responsive patients to routine NACT [25], introduction of a new line chemotherapy could be studied for efficacy, taking into account that continuation of the same regimen has been proven to be inefficient and impose toxicity [17]. A second-line NACT must be conservatively proposed inducing the least harm to the patient that has received previous cycles of chemotherapy. Carboplatin and gemcitabine were proposed as second-line agents being supported by previous studies for their efficacy and least toxicity in breast cancer patients [21–24]. Combination of carboplatin and gemcitabine as first line NACT has also been evaluated and has shown promising effects [31].

Various response rates to neoadjuvant chemotherapy have been reported, however these rates have always been unsatisfactory and different strategies have been evaluated to increase pCR rates in LABC patients. In this study, addition of carboplatin and gemcitabine in clinically non-responsive patients yielded an acceptable pCR rate equal to 32%; though higher than the pCR rate in the control group (29.6%), results did not significantly vary between the arms. Degree of pathologic response evaluated by RCB scoring allows a more detailed understanding of response to NACT. In the total study population, a large number of patients (40.4%) were marked as chemoresistant (RCB III), showing the inefficacy of cytotoxic chemotherapy in either group. Considering the fact that resistance to achieving pCR was mainly attributed to incomplete response in lymph nodes, we may suggest that immune mechanisms may be involved in this resistance pattern and addition of extra cycles of cytotoxic chemotherapy may not be as beneficial as expected. This presumption puts forth the necessity of evaluating probable responsible immune pathways that may oppose to achieving response in the use of cytotoxic agents. While TILs has been associated to better response rates to cytotoxic chemotherapy in more aggressive breast cancer subtypes [20, 32], our results indicate that TILs can help towards better response to NACT in the “intervention” group. T-cell response to tumor growth has been shown to be activated by cytotoxic therapy with different mechanisms [33]. Not only doxorubicin and paclitaxel increase TILs, carboplatin has been found to increase MHC complex class 1 on tumor cells [34]. It is notable that reduction in MHC complex class 1 expression can lead to tumor immune escape. Therefore, it can be postulated that in patients with initial high TILs, addition of carboplatin containing NACT regimens may induce better activation of T-cell mediated response.

Data regarding second line NACT in LABC patients is scarce and approaches to improvement of pCR rate has mainly focused on change of the first line regimen. A similar study concerned with the addition of second-line NACT consumed capecitabine and radiation therapy in non-responders following the same first-line NACT as our study [35]. This study reported a very low pCR rate (4%) compared to our study results. Aside from difference in the second line agents used, the mentioned study also lacked using a control group for the better comparison of the two arms. Another method used to improve response rates partially similar to the design of our study, is the response adjusted sequential therapy that allows the clinician to evaluate response during NACT, and decision of continuing the same regimen or shifting to an alternative can be made according to early response rates. Unsatisfactory results have been reported in previous studies practicing the mentioned protocol. The unsatisfactory results could be however attributed to the selection of chemotherapy agent rather than the method and sequence of their administration. Though in this study, stepwise clinical evaluation of patients led to the omission of several clinically responsive patients from randomization, further chemotherapy and probable adverse effects. In this regard we must further clarify that clinically non-responsive patients were patients with remaining mass either detected with palpation and/or US. However, this remaining mass may not necessarily be malignant (remaining fibrotic/non-malignant tissue following NACT) and this is the reason that the definite evaluation of response is based on pathologic assessment. This explanation justifies the results indicating 29.6% pCR in the “control” group, although no further NACT was used after clinical evaluation of their breast mass.

This study’s results show highest response rates in tissue alone in both “control” and “intervention” groups contrary to a previous report indicating higher response rates in lymph nodes [36]. Nodal response was higher in our carboplatin/gemcitabine recipients although difference was not significant between the groups (Table 2). While relatively high response rates were also observed in lymph nodes in both groups, this study proves the difficulty in achieving pathologic response in lymph nodes compared to tissue in LABC patients and puts forth the poor prognosis of such patients albeit treatment with NACT. Residual nodal involvement following NACT has been associated with poor prognosis in LABC patients [37, 38].

Younger age (< 40) has been introduced as an independent factor in achieving pCR especially in the hormone receptor positive/HER2 negative women [39, 40]. In this study contrary to previous reports, younger age is associated with lower levels of pCR in the carboplatin/gemcitabine recipients (*P* value 0.04). In the control group as relevant to previous studies, younger age was dominant in the pCR patients, although results were insignificant (*P* value > 0.05) (Table 4).

Reports indicating association of BMI with pCR have been variable [41, 42]. Our study results indicate no association between BMI and pCR in either groups or total study population, though higher pCR rate was observed in women with BMI > 25.

Hormone receptor, HER2 and ki67 status have been introduced as prognostic factors in achieving response to NACT. ER negative tumors, HER2 positivity and high ki67 expression of tumors have all been associated with better pCR rates [43–45]. Significance in achieving pCR was evident in different molecular subtypes and contrary to most published data, the HR+/HER2+ subtype showed the highest frequency in pCR cases in both the “intervention” group and total study population (87.5 and 75% respectively) (Table 5). The positive influence of HER2 expression may have overweighed the negative impact of hormone receptor positivity. In other words, HR+/HER2+ subtypes are known to have higher proliferation rates and lower PR expressions of which both could help towards achieving higher pCR rates. Another explanation could be as mentioned in a previous study conveying that addition of cycles of chemotherapy can be more effective in achieving pCR in the hormone receptor positive subtype of breast cancer [46]. In our study, lowest pCR rates were observed in the HR+/HER2- group in accordance with previously published data [47, 48]. Significantly lower extent of ER expressions were observed in the pCR group (43.69% ± 36.3) compared to the non-pCR patients (77.32% ± 20.88), (*P* value = 0.005) relevant to the results of an only published data regarding quantitative assessment of ER expression and its relation with response to NACT [49]. HER2 expression correlated with pCR rates in this study as observed in previous studies [44, 45, 50]. Aside from the effect of targeted therapy against HER2 expression, the high proliferative nature of HER2 positive molecular subtypes can also be an explanation to their higher pCR rates [51]. As an explanation to our results, some reports have also linked HER2 positive tumors with better sensitivity to doxorubicin based chemotherapy [52, 53]. ki67 expression showed significant association with response to NACT in our total study population regardless of chemotherapy agent used (*P* value 0.025). Also mean ki67 values were higher in pCR group compared with non-pCR. Higher proliferation rates observed in tumors with high ki67 expression rationalizes their higher response rates to NACT. Higher tumor grades of breast carcinoma have been also associated with better response rates which is reasonable due to their more rapid growth patterns [42, 54]. This finding was also evident in our study population.

In selection of a second-line chemotherapy regimen, minimal toxicity must be expected. Adverse effects following consumption of carboplatin and/or gemcitabine have been variable. However mostly reported side effects for both can be attributed to bone marrow suppression [31, 55–57] as also seen in our study population. In this study though significant difference was observed in the carboplatin/gemcitabine recipients concerning adverse events, the combined regimen was tolerable for the patients and did not lead to major grade 4 adverse events or hospitalization.

Like every other study, this study is not without limitations. The small number of LABC patients compared to the large number of breast cancer patients detected and also the difficulties of recruiting patients for taking part in clinical trials limited the better gathering of the study population. Follow-up for patients was limited to the time of achieving our study’s endpoint (response to NACT), and survival studies could be included in following researches. As for some trials in the field of oncology, if this study was designed as a non-control based study, by comparing results of the intervention to relevant previously reported data, we assume that the results would have shown more promising impact.

## Conclusion

Although our results may not be conclusive in approving the benefits of using carboplatin/gemcitabine as second line NACT to increase pCR rates, our overall pCR rates in both groups and total study population were appreciable considering our definition for pCR (ypT0/is ypN0) compared with other studies. It may be postulated that activation of immune pathways may be more evident in carboplatin recipients with initial high TILs following anthracycline based chemotherapy. Also the safety observed following the addition of the two agents may support its use; However further studies with larger groups of patients are mandatory in achieving convincing results regarding the efficacy of carboplatin/gemcitabine regimen as a second-line NACT in LABC patients.

## Data Availability

The data that support the findings of this study are available from the corresponding author upon reasonable request.

## Abbreviations

NACT: Neoadjuvant chemotherapy
LABC: Locally advanced breast cancer
pCR: Pathologic complete response
HER2: Human epidermal growth factor receptor 2
HR: Hormone receptor
TIL: Tumor infiltrating lymphocyte
DCIS: Ductal carcinoma insitu
FDA: Food and Drug Administration
RCB: Residual cancer burden
RCT: Randomized controlled trial
LVEF: Left ventricular ejection fraction
US: Ultra sound
IRCT: Iranian registry of clinical trials
CT: Computed tomography
MRI: Magnetic resonance imaging
IHC: Immunohistochemistry
HRP: Horseradish protein
TNBC: Triple negative breast cancer
AJCC: American Joint Committee on Cancer
CTCAE: Common Terminology Criteria for Adverse Events
MHC: Major histocompatibility complex
